# Fenofibrate attenuates doxorubicin-induced cardiac dysfunction in mice via activating the eNOS/EPC pathway

**DOI:** 10.1038/s41598-021-80984-4

**Published:** 2021-01-13

**Authors:** Wen-Pin Huang, Wei-Hsian Yin, Jia-Shiong Chen, Po-Hsun Huang, Jaw-Wen Chen, Shing-Jong Lin

**Affiliations:** 1 Division of Cardiology, Cheng-Hsin Rehabilitation Medical Centre, Taipei, Taiwan; 2 Division of Cardiology, Department of Medicine, Taipei Veterans General Hospital, Taipei, Taiwan; 3 Department of Critical Care Medicine, Taipei Veterans General Hospital, 112, No. 201, Sec. 2, Shih-Pai Road, Taipei, Taiwan; 4 Department of Medical Research, Taipei Veterans General Hospital, Taipei, Taiwan; 5 Healthcare and Management Center, Taipei Veterans General Hospital, Taipei, Taiwan; 6 Institute of Pharmacology, National Yang-Ming University, Taipei, Taiwan; 7 Institute of Clinical Medicine, National Yang-Ming University, Taipei, Taiwan; 8 Cardiovascular Research Center, National Yang-Ming University, Taipei, Taiwan

**Keywords:** Biochemistry, Cell biology, Chemical biology, Drug discovery

## Abstract

Endothelial progenitor cells (EPCs) improve endothelial impairment, which in turn restores endothelial function in patients with heart failure (HF). In the present study, we tested whether fenofibrate, with its anti-inflammatory and vasoprotective effects, could improve myocardial function by activating EPCs through the eNOS pathway in a doxorubicin (DOX)-induced cardiomyopathy mouse model. Wild-type mice were divided into 4 groups and treated with vehicle, DOX + saline, DOX + fenofibrate, and DOX + fenofibrate + L-NAME (N(ω)-nitro-L-arginine methyl ester). DOX-induced cardiac atrophy, myocardial dysfunction, the number of circulating EPCs and tissue inflammation were analyzed. Mice in the DOX + fenofibrate group had more circulating EPCs than those in the DOX + saline group (2% versus 0.5% of total events, respectively) after 4 weeks of treatment with fenofibrate. In addition, the inhibition of eNOS by L-NAME in vivo further abolished the fenofibrate-induced suppression of DOX-induced cardiotoxic effects. Protein assays revealed that, after DOX treatment, the differential expression of MMP-2 (matrix metalloproteinase-2), MMP-9 (matrix metalloproteinase-9), TNF-α (tumor necrosis factor-α), and NT-pro-BNP (N-terminal pro-B-type natriuretic peptide) between saline- and DOX-treated mice was involved in the progression of HF. Mechanistically, fenofibrate promotes Akt/eNOS and VEGF (vascular endothelial growth factor), which results in the activation of EPC pathways, thereby ameliorating DOX-induced cardiac toxicity.

## Introduction

The prevalence of chronic heart failure is approximately 1–2% of the adult population in developed countries and rises to ≥ 10% among people > 70 years of age^1^. Chronic heart failure is a consequence of cardiac remodeling processes that are induced by various types of heart diseases, such as myocardial infarction, chronic hypertension, or exposure to toxic agents^2^. The anthracycline doxorubicin (DOX) is widely used as an effective antitumor drug, but its clinical use is limited by cardiotoxicity that leads to congestive heart failure (CHF)^3^. Although a variety of small molecular compounds with antioxidant activity, such as resveratrol, isorhamnetin, diosgenin and acute exercise have been used in an attempt to protect the heart against DOX-induced cardiotoxicity, treatment to prevent short- and long-term DOX-induced cardiac damage remains limited^4–7^.

Endothelial progenitor cells (EPCs) have angiogenic capabilities and are the leading players in endogenous repair mechanisms that counteract endothelial dysfunction^8^. HF patients with failing circulation are characterized not only by depressed cardiac function but also by endothelial dysfunction^9^. Circulating EPCs and CD34 + cells are negatively correlated with classification in the HF functional class by the New York Heart Association^9,10^. Clinical studies also reported a significant improvement in angina frequency and exercise tolerance with the intramyocardial transplantation of autologous CD34 + cells in patients with refractory angina^11–13^. Peroxisome proliferator-activated receptor α (PPARα), which was the first identified PPAR isoform, is a target for various long-chain fatty acids and is predominantly expressed in tissues exhibiting high rates of fatty acid catabolism^14^. PPARα deficiency may lead to the impaired functional capacity of the heart^15^. Recent studies demonstrated the numerous pleiotropic effects of fenofibrate, a PPARα activator, on the heart that afford direct myocardial protection in addition to the lipid-lowering effects of fenofibrate^16,17^. Short-term treatment with fenofibrate improved vascular endothelial function in healthy, normal lipidemic, middle-aged/older adults by reducing oxidative stress and increasing eNOS activation^18^. Our study also showed that fenofibrate exerted beneficial effects in patients with systolic HF and that activated PPARα attenuated ET-1-induced cardiomyocyte hypertrophy^19,20^. Although fenofibrate exerted a protective effect against vascular dysfunction by its anti-inflammatory properties, we also observed that fenofibrate, a PPARα agonist, stimulated angiogenesis by improving endothelial precursor cell function, mobilization, and homing of endothelial progenitor cells^21,22^. We suspect that fenofibrate could have a protective mechanism in the cardiovascular system independent of its anti-inflammatory properties. CHF is common in elderly individuals in developed countries, and the chemotherapy drug doxorubicin is used in the treatment of breast cancer patients. The proposed principal mechanisms of doxorubicin cardiotoxicity are significant myelosuppression, induced oxidative stress, and promote cellular apoptosis of cardiomyocytes^23–25^. Whether fenofibrate is involved in the pathogenesis of DOX-associated heart toxicity is unclear. Thus, we aimed to investigate whether fenofibrate could have a protective effect against DOX-associated myocardial damage and to clarify the role of EPCs in DOX-induced heart toxicity in vivo and in vitro.

## Materials and methods

### Animal experiments

**Figure 1:**
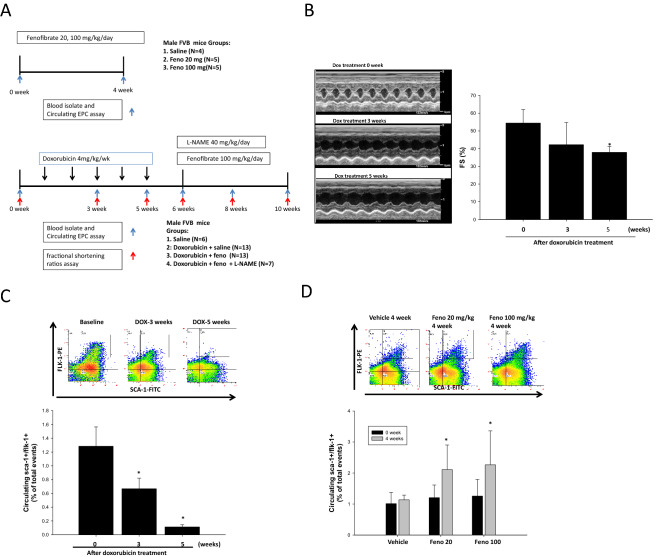
DOX-induced cardiac dysfunction and decrease in the number of circulating EPCs in FBV mice. (**A**) The DOX, fenofibrate, and L-NAME treatment protocols used in this animal study. (**B**) Quantitative analysis of left ventricular short axis fractional shortening (LVFS) after DOX IV injection, which was determined by echocardiography in FBV mice at 0, 3, and 5 weeks. (**C**) The number of circulating EPCs (defined as Sca-1 + /Flk-1 + cells) after DOX IV injection was determined by flow cytometry in FBV mice at 0, 3, and 5 weeks. Doxorubicin n = 33. (**D**) EPC (defined as Sca-1 + /Flk-1 + cells) mobilization after the oral administration of fenofibrate (20 and 100 mg/kg) was determined by flow cytometry in FBV mice at 4 weeks. *p < 0.05 vs. saline. Experiments was with n = 4–5/group. One-way ANOVA.

All FVB/NJ mice were purchased from the (BioLASCO Taiwan Co., Ltd.), 8 weeks of age and weighing approximately 24 g each. First part as Fig. 1A were administered group1) saline, 2) fenofibrate (Santa Cruz Biotechnology, Santa Cruz, CA, USA) orally in feed for 4 weeks (20 mg kg-1 d-1), 3) fenofibrate orally in feed for 4 weeks 100 mg kg^−1^ d^−1^^26,27^. Second part as Fig. 1A mice were administered 1) saline, 2) DOX 4 mg kg-1 week-1, (Sigma, USA) by intravenous tail injection for five cycles (single dose/week, cumulative dose: 20 mg/kg) to establish cardiac dysfunction^28^. After 3 and 5 weeks, ventricular structure and function were assessed by echocardiography before the animals were randomly divided into three groups that received Followed by treatment 1) saline, 2) fenofibrate orally in feed for 4 weeks (100 mg kg-1 d-1), 3) fenofibrate + L-NAME (Santa Cruz Biotechnology, Santa Cruz, CA, USA) at a dosage of 40 mg kg^−1^ d^−1^^29,30^. (IACUC of Taipei Veterans General Hospital: approval No. 2018-009). We provided a flow chart to show the number of animals in this study (Supplemental Fig. 1).

Fenofibrate solution in DMSO (Sigma-Aldrich, St Louis, MO, US) was administered as a suspension in water, which was used as a vehicle (5% MDSO). This vehicle was used as a negative control. One hundred microliters of fenofibrate and vehicle were administered daily by oral feeding gavage for 4 weeks. After 4 weeks, ventricular structure and function were assessed by echocardiography (Fig. 1A). All experimental animal procedures were approved by the Animal Care and Use Committee of the National Medical University and performed in accordance with the Guide for the Care and Use of Laboratory Animals (NIH publication No. 85–23, National Academy Press, Washington, DC, USA, revised 1996). In correlation assay, only including doxorubicin treated mice.

At the end of the treatment period, the mice were anesthetized with an intraperitoneal injection of avertin (250 µl/25 g, 240 mg/kg)). Then, blood samples were collected from the submandibular vein, centrifuged at 3000 rpm for 10 min to separate the serum, and stored at − 80 °C for biomarker analysis. The heart tissues were isolated, weighed, washed with PBS, and separated into two samples. One was used for immunohistochemistry (IHC) and fixed in 4% formalin. The other was stored at − 80 °C for a Western blotting assay.

### EPC isolation and identification

**Figure 2:**
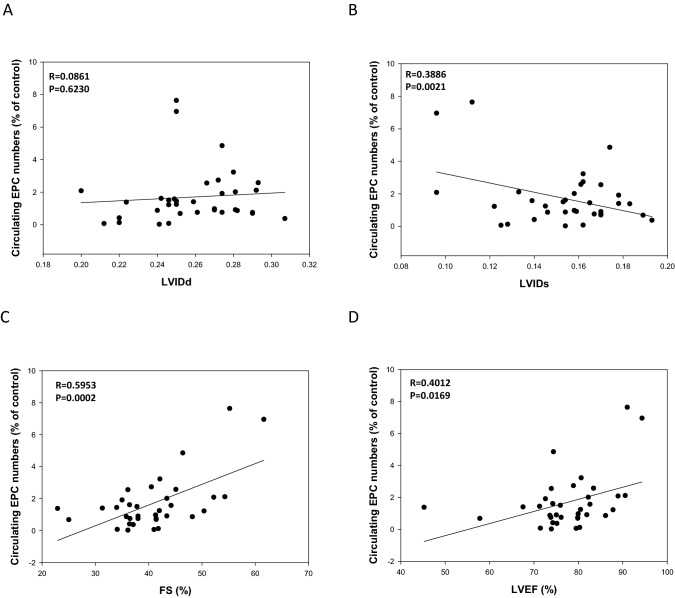
Correlations among circulating EPC cell numbers (%) and LVIDd levels (**A**); circulating EPC cell numbers (%) and LVIDs levels (**B**); and circulating EPC cell numbers (%) and LVFS levels (**C**), and LVEF levels (**D**) in FBV mice after DOX IV injection at 5 weeks (n = 33).

To investigate the effects of fenofibrate on EPC mobilization in the circulating peripheral blood (PB) in response to DOX, a fluorescence-activated cell sorting (FACS) Caliber flow cytometer (Becton Dickinson, San Jose, CA, USA) was used to assess EPC mobilization. First, 100 μL of peripheral blood (PB) was collected from the cheeks of the FVB/NJ mice. Then, the cells were lysed in red blood cells (RBC) were lysed by using lysis buffer (BD Pharmingen, NJ, USA) to harvest mononuclear cells, which were incubated with 2 µl of fluorescein isothiocyanate (FITC)-labeled anti-mouse Sca-1 (eBioscience, San Diego, CA, USA) antibody and 2 µl of phycoerythrin (PE)-labeled anti-mouse Flk-1 (VEGFR-2, eBioscience) antibody. We also identity other surface antigen co-expressed on sca1 + /flk1 + cells including, 2 µl of peridinin chlorophyll protein (PerCP) anti-mouse CD117 (c-Kit) antibody (BioLegend, San Diego, CA), 2 µl of PerCP anti-mouse CD4 antibody (BioLegend), 2 µl of PerCP anti-mouse CD8 antibody (BioLegend), 2 µl of PerCP anti-mouse Ly6C antibody (BioLegend), 2 µl of allophycocyanin (APC) anti-mouse CD135 antibody (BioLegend), 2 µl of APC anti-mouse CD16/32 antibody (BioLegend), 2 µl of APC anti-mouse CD3 antibody (BioLegend), 2 µl of APC anti-mouse CD11b antibody (BioLegend) in a final volume of 100 µl PBS at 4 °C for 30 min (Supplemental data and S-Fig. 2). Isotype-identical antibodies served as controls (Becton Dickinson, Franklin Lakes, NJ, USA). After incubation for 30 min, the cells were washed with phosphate-buffered saline (PBS) and analyzed. Each analysis, we used the total events (n > 50,000) as 100%, and determined the circulating EPC numbers as the percentage of the double positive Sca-1/Flk-1 cells from the mononuclear fractions.

### Western blot analysis

The hearts were lysed in lysis buffer (62.5 mM Tris–HCl, 2% SDS, 10% glycerol, 0.5 mM PMSF, and 2 μg/mL aprotinin, pepstatin, and leupeptin) as previously described^31^. Proteins in the cell lysates were separated using 10% SDS–polyacrylamide gel electrophoresis followed by transfer to PVDF membranes. Membranes were probed with monoclonal antibodies against p-eNOS, eNOS (Upstate Biotechnology, Lake Placid, NY, USA), β-actin (Chemicon), p-Akt, Akt (Cell Signaling Technology, Beverly, MA, USA), MMP-2, MMP-9 (Merck Millipore, Billerica, MA, USA), VEGF, and SDF-1 (Abbiotec, La Jolla, CA, USA). Bound antibodies were visualized using chemiluminescence detection reagents (Merck Millipore, Billerica, MA, USA). Protein band densitometry was conducted using ImageQuant software (Promega, Madison, WI, USA).

### Measurement of cardiac function

Transthoracic echocardiographic analysis was performed as previously described^32^. M-mode images of the left ventricle were recorded after the animals were anesthetized with avertin. The following parameters were measured to evaluate cardiac structure and function: left ventricular end-diastolic diameter (LVIDd), left ventricular ejection fraction (LVEF), and left ventricular short axis fractional shortening (LVFS). All parameters were assessed using an average of three heart beats.

### Determination of plasma biomarker concentrations

Blood samples were collected from the hearts after the animals were euthanized. The levels of MMP2, MMP-9, TNF-α, VEGF, adiponectin, BNP, and NT-pro-BNP in circulation were determined by ELISA kits. Mouse MMP-9, VEGF, and adiponectin ELISA kits were purchased from R&D Systems (Minneapolis, MN), mouse TNF-α ELISA kits were purchased from Invitrogen (Carlsbad, CA), and mouse BNP and NT-pro-BNP ELISA kits were purchased from MyBioSource (San Diego, CA, USA). The procedures were carried out according to the manufacturers’ instructions.

### Hematoxylin and eosin (HE) staining

The left ventricle (LV) myocardium was fixed in 4% formalin, cut transversely, embedded in paraffin, and stained with hematoxylin and eosin. Five randomly selected fields per section were analyzed. Digital photographs were taken using a high-resolution digital image analysis system (QwinV.3, Leica, Germany).

### Statistical analysis

Continuous variables are expressed as the means ± SDs, and categorical variables are presented as frequencies and percentages. Comparisons of biochemical characteristics between two groups were analyzed using a two-tailed Student’s t test. Comparisons of animal study characteristics differences between multiple groups were analyzed using one way ANOVA followed by Dunnett’s test with SPSS (version 17.0; SPSS Inc., Chicago, IL, USA), and a p value < 0.05 indicated statistical significance.

## Results

### DOX-induced cardiac dysfunction in mice and reduced circulating EPC cell number

As shown in Fig. 1A, DOX-induced cardiac dysfunction was induced in FBV mice injected five times with DOX (4 mg mg kg-1 week-1), and cardiac function was evaluated by echocardiography. No deaths were observed in the DOX-treated groups. Figure 1B shows the quantitative analysis of M-mode echocardiograms after DOX administration in mice. DOX treatment caused a pronounced reduction in cardiac contractility compared to that observed before DOX treatment, reflected by fractional shortening (FS %) in the mice (baseline at 0 week: 54.4 ± 7.6, after 5 weeks of DOX treatment: 37.8 ± 3.3). To clarify whether suppressed EPC numbers were associated with DOX-induced cardiac dysfunction, circulating EPC numbers were evaluated by flow cytometry after 2 weeks of DOX injection. Figure 1C shows that fewer circulating EPCs were observed following DOX administration in FBV mice than before DOX treatment (baseline at 0 weeks: 1.28 ± 0.28%, after 2 weeks of DOX treatment: 0.66 ± 0.15%, after 5 weeks of DOX treatment: 0.11 ± 0.03 of total events). In addition, we determined the effect of fenofibrate on the number of circulating EPCs. As depicted in Fig. 1D, there were significantly more circulating EPCs in fenofibrate-treated mice than in vehicle-treated FBV mice after 4 weeks of fenofibrate treatment (EPC at 4th week, Vehicle vs. Feno 100, 1.0% vs. 3.8%, p < 0.05; Vehicle vs. Feno 20, 1.0% vs. 2.1%).

Furthermore, to evaluate the correlation between EPC number and cardiac function, as presented in Fig. 2B**,** we examined LVIDs measurements and found that they were significantly inversely correlated with the number of circulating EPC cells (r = 0.38, P = 0.002), whereas there was no significant correlation with LVIDd measurements (r = 0.08, P = 0.6, Fig. 2A). There was a significant correlation between the number of circulating EPCs and LVFS (r = 0.59, P = 0.0002, Fig. 2C), whereas there was significant correlation with LVEF measurements (r = 0.4012, P = 0.0169 Fig. 2D).These findings indicated that treatment with fenofibrate ameliorated DOX-induced cardiac dysfunction and that decreased numbers of circulating EPCs may be involved in DOX-induced cardiac dysfunction.

### DOX-induced reduction in circulating EPC cell numbers and cardiac dysfunction were recovered by fenofibrate treatment in mice

**Figure 3:**
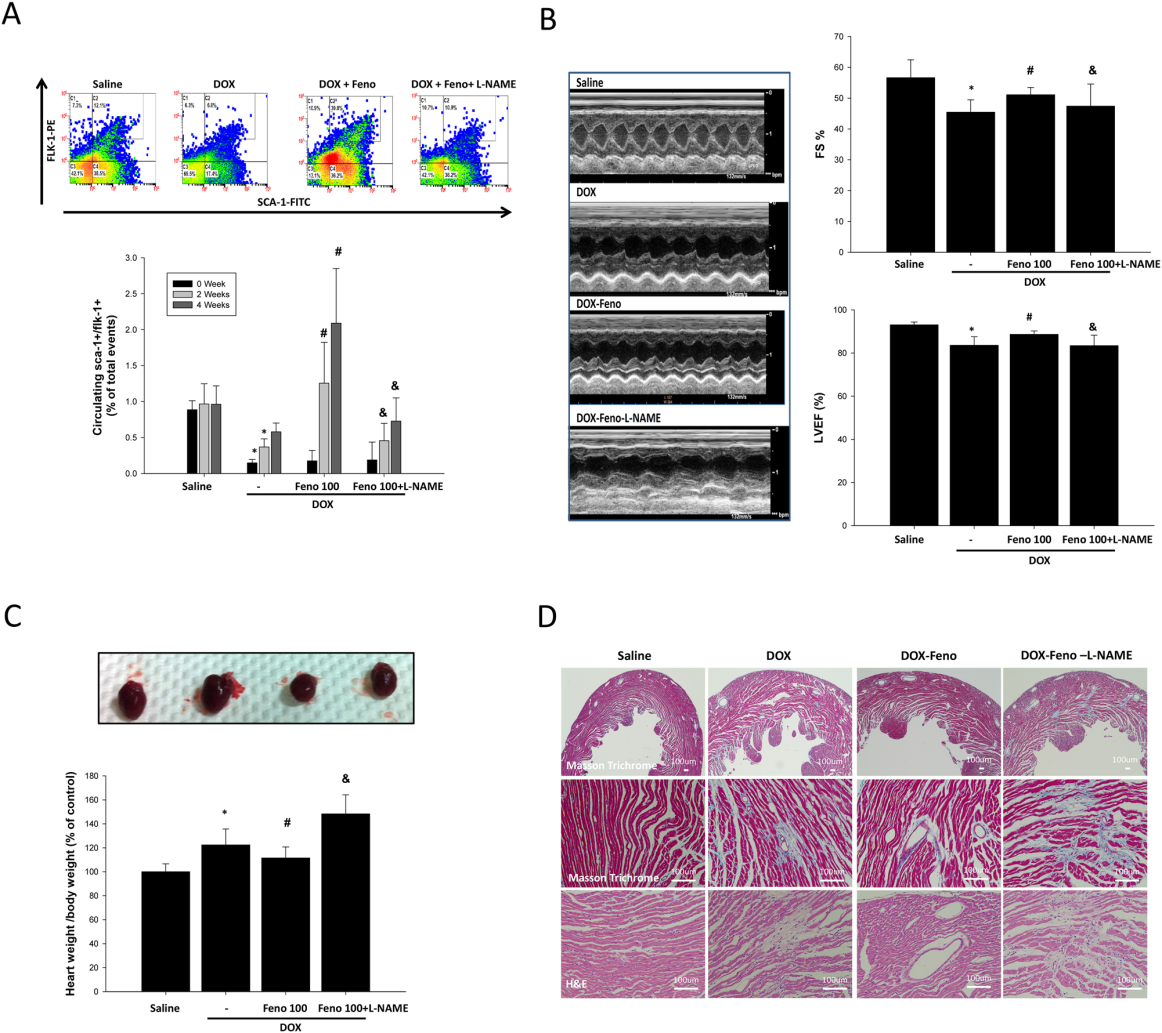
Effect of fenofibrate on cardiac dysfunction in doxorubicin-treated wild-type FBV mice. (**A**) FBV mice after the administration of saline, fenofibrate, or fenofibrate + L-NAME after DOX treatment. Fenofibrate significantly improved EPC mobilization after DOX treatment, and fenofibrate reversed the decrease in EPC cell numbers with L-NAME treatment. (**B**) M-mode echocardiographic images of mice treated with saline, fenofibrate, and fenofibrate + L-NAME after DOX treatment. Quantitative analysis of LVFS in mice treated with saline, fenofibrate, and fenofibrate + L-NAME after DOX treatment. (**C**) Heart weights and body weight ratios of mice treated with saline, fenofibrate, and fenofibrate + L-NAME after DOX treatment. *p < 0.05 versus saline; #*p* < 0.05 versus DOX alone; and *p* < 0.05 versus DOX + fenofibrate. One-way ANOVA. (**D**) Masson’s trichrome staining was performed on heart sections from each of the groups: fenofibrate and fenofibrate + L-NAME after DOX treatment, N = 3–4/group. Experiments were n = 6–13/group.

As shown in Fig. 3A, the DOX-induced reduction in the number of circulating EPCs increased following 2 and 4 weeks of fenofibrate treatment, and this effect was inhibited by L-NAME. In addition, DOX treatment caused a pronounced reduction in cardiac contractility reflected by fractional shortening and left ventricular ejection fraction (LVEF), which increased following treatment with fenofibrate, and this effect was blocked by L-NAME (Fig. 3B). As shown in Fig. 3C and Table 1, similar results were observed for heart/body weight ratios (baseline in healthy controls: 100 ± 6.6%; 5 weeks of DOX treatment followed by 4 weeks of saline treatment, 122.4 ± 13.3%; 5 weeks of DOX treatment followed by 4 weeks of fenofibrate treatment: 111.4 ± 9.3%; 5 weeks of DOX treatment followed by 4 weeks of fenofibrate + L-NAME: 148.2 ± 15.9%). This fenofibrate-induced protective effect was virtually absent in the L-NAME treatment group. To further elucidate the effect of fenofibrate on interstitial cardiac fibrosis and vascular fibrosis in DOX-treated mice, Masson’s trichrome staining was performed on myocardial sections from each of the groups. Representative photomicrographs showing Masson’s trichrome staining of interstitial fibrosis are shown in Fig. 3D. These results suggested that circulating EPC numbers were correlated with the protective effect of fenofibrate against the progression of DOX-induced cardiac dysfunction.

**Table 1:** Serum parameters in wild-type FVB/NJ mice after 9 weeks of dietary treatment.

List	Vehicle	DOX	DOX + Feno	DOX + Feno + L-NAME
Body weight, (g)	33.2 ± 2	29.1 ± 3.7	22.6 ± 3.7^#^	20.5 ± 2.1
Heart weight, (mg)	104 ± 9	113 ± 16*	83 ± 16^#^	96 ± 12
MMP-9, (ng/mL)	7.1 ± 1	14 ± 5.8*	10.9 ± 5.9^#^	16.1 ± 3.2^&^
TNF-α, (pg/mL)	10.3 ± 6	16 ± 7*	12.3 ± 6.6	19.5 ± 4.1
VEGF, (pg/mL)	5 ± 0.8	6.3 ± 0.9	5.2 ± 0.6^#^	6.4 ± 1.3^&^
ADP, (μg/mL)	2.6 ± 0.2	3.2 ± 0.4	2.5 ± 0.3^#^	3.4 ± 0.6^&^
BNP, (pg/mL)	137 ± 40	195 ± 24*	156 ± 29^#^	175 ± 5
NT-pro-BNP (pg/mL)	1107 ± 79	1527 ± 91*	1230 ± 150^#^	1147 ± 92^&^

Values are the mean ± standard deviation (SD). *P < 0.05, for DOX versus vehicle; ^#^P < 0.05, for DOX + Feno versus DOX, ^&^P < 0.05, for DOX + Feno + L-NAME versus DOX.

DOX: doxorubicin, Feno: fenofibrate, L-NAME: N^G^-nitro-L-arginine methyl ester, MMP-9: matrix metallopeptidase 9; TNF-α: tumor necrosis factor alpha, VEGF: vascular endothelial growth factor, ADP: adiponectin, BNP: brain natriuretic peptide, NT Pro-BNP: N-terminal pro-brain natriuretic peptide.

### Effect of fenofibrate on endothelial activation signaling in mice

**Figure 4:**
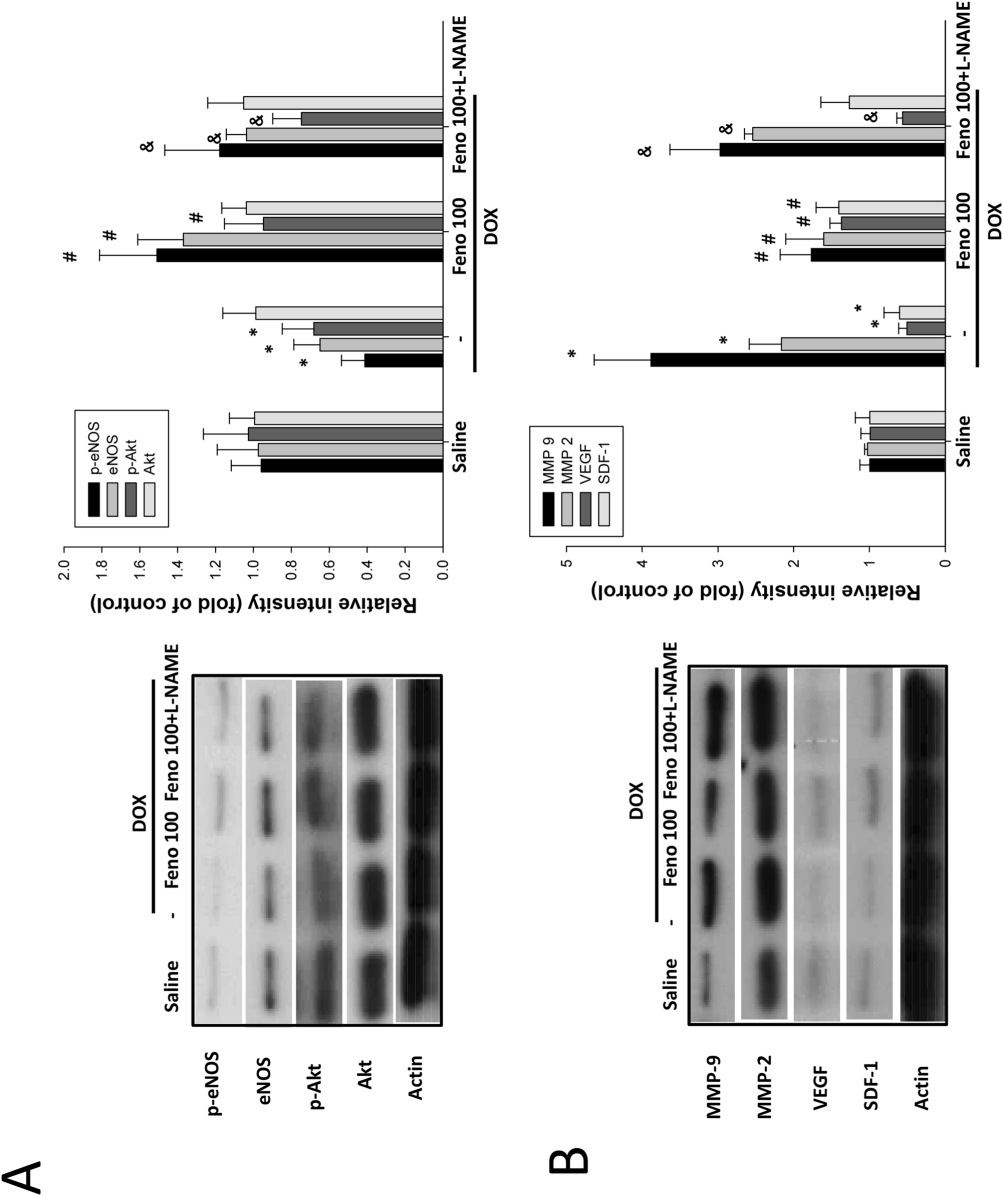
Effect of fenofibrate on endothelial activation marker expression in the hearts of FBV mice after DOX treatment. (**A**) Protein expression of endothelial activation signaling pathways. The phosphorylation of Akt/eNOS was measured by Western blot and normalized to the phosphorylation of the control group. (**B**) The protein expression levels of the inflammation and angiogenesis proteins MMP-9, MMP-2, VEGF, and SDF-1 were tested and normalized to the expression levels in the control group (mean ± SDs, n = 3). *p < 0.05 versus saline; #*p* < 0.05 versus DOX alone; and *p* < 0.05 versus DOX + fenofibrate. One-way ANOVA.

To further determine whether Akt/eNOS signaling pathways were involved in the mechanism of fenofibrate cardioprotection against DOX, we measured the phosphorylation of eNOS and Akt and found that it was attenuated by DOX, as shown in Fig. 4A. Additionally, the phosphorylation of both eNOS and Akt increased following treatment with fenofibrate, and this effect was lessened by L-NAME treatment. Similar results were also observed for the accumulation of eNOS, which demonstrated that fenofibrate reversed the DOX-induced suppression of eNOS activation, and this effect of fenofibrate was attenuated by L-NAME treatment. Taken together, the findings above suggest that the protective effect of fenofibrate is attenuated by the DOX-induced blockage of the endothelial lineage activation pathway, resulting in insufficient EPC recruitment to the heart and cardiac dysfunction.

We also evaluated inflammation and angiogenesis-associated protein expression. Figure 4B and Table 1 show that the levels of both MMP-9 and MMP-2 were significantly increased in response to DOX compared to those in control cells, and fenofibrate attenuated DOX-induced MMP-2 and MMP-9 accumulation in the heart. The protective effect of fenofibrate was suppressed by L-NAME treatment. Moreover, similar effects were also observed for the levels of MMP-9 and TNF-α, as fenofibrate attenuated the DOX-induced increase in MMP-9 and TNF-α accumulation, but this effect was lessened by L-NAME treatment. These results suggested that fenofibrate attenuated DOX-induced heart inflammation in mice by eNOS activation. Finally, similar results were also seen for both the VEGF and SDF-1 proteins in response to DOX. Fenofibrate reversed the DOX-induced suppression of VEGF and SDF-1 accumulation in the heart, but this effect was reduced by L-NAME treatment. However, L-NAME had no effect on the fenofibrate-induced reversal of SDF-1 suppression, which suggests that fenofibrate is a PPAR-α agonist that triggers SDF-1 expression independent of the NO pathway. These results suggest that the activation of NO signaling was crucial for the protective effect of fenofibrate against the progression of DOX-induced cardiac dysfunction.

## Discussion

Here, we showed that treatment with fenofibrate led to cardioprotection against doxorubicin in mice. We showed that a fenofibrate-mediated increase in the circulation of EPCs and the activation of cardiac NO and angiogenesis factors, which were associated with an attenuated inflammatory response, contributed to improved LV function. In agreement with previous studies and in line with findings derived from human studies, the use of doxorubicin in our model led to severely reduced LV systolic and diastolic function, which consequently resulted in cardiac dysfunction^3,33^. Treatment with fenofibrate improved LV function under these conditions. These findings are in agreement with a previous study that demonstrated that fenofibrate attenuated daunorubicin-induced cardiac toxicity^27^. Daunorubicin and doxorubicin show some differences in their clinically approved indications, especially the use of doxorubicin to treat solid tumors^34^. Solid tumors are not easy to treat, so the treatment period is longer, which increases the risk of myocardial toxicity, and there is currently no drug to treat this side effect. We are interested in looking for a drug that can attenuate the risk of myocardial toxicity with the use of doxorubicin for anticancer treatment.

DOX is one of the most important anticancer agents. However, the clinical use of DOX is limited by its cardiotoxicity. Although the precise mechanisms whereby DOX induces myocardial injury have not been fully elucidated, it is widely accepted that DOX induces cardiac injury via several mechanisms, including the induction of proinflammatory cytokines, the generation of free radicals, the promotion of apoptotic cell death, and the suppression of EPC mobilization and function, which are typical changes observed in DOX-induced heart failure^3,35^. In the present study, 5 weeks of DOX treatment induced heart hypertrophy, increased LVIDs measurements, and reduced fractional shortening (FS) % in mice compared to those observed in mice without DOX treatment. These results suggested that doxorubicin led to cardiac dysfunction in our model. Furthermore, we also clarified that DOX attenuated the number of circulating EPCs in a time-dependent manner in mice and that the number of circulating EPCs was negatively correlated with LVIDs measurements and positively correlated with the recovery of EPC numbers, which may contribute to the reduction of LVIDs measurements, indicating that the recovered EPC number may contribute to a reduction in the LVIDs. Similar results were also found for the mobilization of EPCs by granulocyte colony-stimulating factor (G-CSF), which effectively improved cardiac function and lessened ventricular remodeling, suggesting that EPC mobilization contributed to improvements in cardiac function^36^. Treatment with fenofibrate significantly improved the suppression of cardiac function and increased the mobilization of EPC numbers compared to those in mice without fenofibrate treatment, and this effect was abolished when L-NAME treatment was included. These results suggested that circulating EPCs play a crucial role in DOX-induced cardiotoxicity. Our results are in agreement with previous study demonstrating that erythropoietin (EPO)-induced enhancement in the number of EPC and their function against myocardial dysfunction were induced by DOX^35^.

It is known that EPC numbers are insufficient in the progression of severe HF^10^. In previous studies, increased the number of EPCs by pharmacologically or through cell transplantation effectively improve the cardiac functions in patients with heart failure in animal and human clinical trials^35,37^. Therefore, the activation of EPCs may be a therapeutic strategy to attenuate the progression of HF. In the present study, EPC activation signaling factors were assayed in serum and heart tissue. The results showed that SDF-1 and VEGF protein levels were increased in heart tissue by fenofibrate treatment compared to those observed without fenofibrate treatment. Similar results were also found for the phosphorylation of eNOS/Akt signaling components in heart tissue. These results suggested that the DOX-induced suppression of EPC activation was reversed by fenofibrate-mediated NO activation signaling and angiogenesis factors. Our results agree with previous results demonstrated that PPARα regulated mobilization and homing of EPCs through the HIF-1α/SDF-1 pathway and fenofibrate treatment restored EPC function by increased NO production in EPCs of diabetic mice^21,22^.

TNF-α, MMP-2, and MMP-9 act on inflammatory processes, are upregulated during heart failure (HF), and influence ventricular remodeling. Our results showed that mice treated with DOX had higher serum levels of TNF-α and MMP-9 than untreated mice. Similar results were also found in heart tissues. These findings agree with a previous study showing that the elevated expression of MMP-9 was associated with collagen maturation in heart failure, demonstrating the important role of these enzymes in fibrosis through collagen configuration, activation, and deposition^38^. In addition, our results showed that the DOX-induced accumulation of TNF-α, MMP-2, and MMP-9 was attenuated by fenofibrate in mice, and L-NAME abolished this effect (Table 1). These results suggested that NO activation is involved in the fenofibrate-induced suppression of TNF-α, MMP-2, and MMP-9 induction in mice. These findings agree with previous studies showing that fibrate increased the transcriptional activities of PPARα and decreased the transactivation of nuclear transcription factor NF-κB and that fenofibrate activated eNOS and increased NO bioavailability, which in turn suppressed MMP-2^39,40^. MMP-9, a well-recognized mediator of adverse ventricular fibrosis and subsequent remodeling, was evaluated to establish the relationship between fenofibrate-activated EPCs and blunted fibrosis in DOX-induced cardiotoxicity.

The role of adiponectin in the progression of heart failure is controversial. Adiponectin deficiency resulted in increased DOX-induced cardiotoxicity^41^. Increased adiponectin levels were correlated with cardiac performance^42^. However, CHF patients with diastolic heart failure had decreased circulating adiponectin levels compared with those in age-matched normal controls^43^. It is possible that adiponectin adopts a two‐faced character in HF. In the early stage of HF, the increase in adiponectin production may be part of a compensatory mechanism to protect the heart. In our study, a DOX-induced increase in adiponectin was observed. Fenofibrate attenuated this DOX-induced increase in adiponectin, which was abolished with L-NAME treatment. In DOX-induced cardiotoxicity, increasing adiponectin was a compensatory mechanism to protect the heart. In this study, L- NAME abolished the effect of fenofibrate on DOX-induced cardiotoxicity, which accompanied increased adiponectin levels. These data suggested that fenofibrate had a minor effect on the increase in adiponectin in DOX-triggered cardiotoxicity. In addition, a previous study reported that the level of pro-BNP was increased by chemotherapy^44^. In our study, DOX-induced increases in circulating BNP and pro-BNP were also attenuated by fenofibrate, and this effect was abolished by L-NAME. These data suggested that fenofibrate reversed DOX-induced cardiotoxicity through the NO activation pathway.

Heart size is positively correlated with body weight. In the present study, we noted that the group treated with fenofibrate for 4 weeks lost a significant amount of body weight. We were concerned about whether fenofibrate suppressed heart size by reducing body weight. Therefore, we carefully analyzed heart M-mode echocardiograms, and we found that fenofibrate significantly reversed DOX-impaired fractional shortening (FS) %, and L-NAME abolished this effect as well as changes in heart size despite for the mice exhibiting similar decreases in body weight. Previous reports showed that alterations in myocardial energy metabolism induced by the anti-cancer drug doxorubicin. It is primarily associated with the increased production of reactive oxygen species (ROS), apoptosis of cardiac cells, mitochondrial damage, and impairment of cardiac energy metabolism^45^. In addition, it has been reported that fenofibrate reduced body weight by reducing serum triglyceride levels^46,47^. Therefore, we suggest that the energy production (especially in mitochondrial) of mice may be impaired after DOX administration. When Fenofibrate is administered after DOX administration, the energy production may be insufficient and the result of a significant weight loss will be observed. As this finding was reported with the long-term treatment of mice with fenofibrate, it was possibly related to the reduced body weight and improved heart function caused by treatment with fenofibrate and not due to the reduction in body weight. Taking all of the above findings together, we suggest that fenofibrate attenuated DOX-induced cardiotoxicity and concomitantly reduced body weight.

There were two limitations to this study. First, we could not rule out the involvement of the AMPK activation pathway in the protective effects of fenofibrate. Although adiponectin levels did not change significantly under fenofibrate treatment, fenofibrate activates eNOS by activating the AMPK pathway^48,49^. Therefore, a fenofibrate-triggered increase in eNOS-mediated EPC function may be a possible mechanistic explanation for its protective effects in DOX-induced cardiotoxicity. A second limitation of this study was the lack of allogeneic EPCs to serve as control samples. For clinical applications, small molecular drugs are easily available, and we used a pharmacological strategy to activate EPC mobilization under DOX challenge. However, it is difficult to explain the real mechanism through which the activation of EPCs reversed DOX-induced cardiotoxicity. Therefore, the transplantation of EPCs to supplement the decreased EPC numbers in mice following treatment with DOX is a direct strategy to prove this hypothesis.

## Conclusion

This study demonstrated the potent relevant efficacy of fenofibrate therapy in mice with DOX-induced cardiotoxicity. Fenofibrate restored the fractional shortening (FS)%, circulating EPCs, and VEGF levels altered following DOX treatment to those observed in control mice. As abnormalities in the heart function of mice with CV disease are highly related to MMPs, TNF-alpha, and pro-BNP, targeting EPC function is a significant therapeutic strategy. Together, these findings offer a new mechanism of action underlying clinically relevant responses to the use of fenofibrate in CV diseases.

### Ethics approval and consent to participate

IACUC of Taipei Veterans General Hospital: approval No. 2018-009, and complied with the Guide for the Care and Use of Laboratory Animals.

## Supplementary Information

Supplementary Informatipon 1.

Supplementary Informatipon 2.

## Data Availability

Supplementary materials can be found at https://jbiomedsci.biomedcentral.com/.

## References

[1] Ponikowski P (2016). 2016 ESC Guidelines for the diagnosis and treatment of acute and chronic heart failure: The Task Force for the diagnosis and treatment of acute and chronic heart failure of the European Society of Cardiology (ESC). Developed with the special contribution of the Heart Failure Association (HFA) of the ESC. Eur. J. Heart Fail..

[2] Azevedo PS, Polegato BF, Minicucci MF, Paiva SA, Zornoff LA (2016). Cardiac remodeling: concepts, clinical impact, pathophysiological mechanisms and pharmacologic treatment. Arq. Bras. Cardiol..

[3] Mitry MA, Edwards JG (2016). Doxorubicin induced heart failure: Phenotype and molecular mechanisms. Int. J. Cardiol. Heart Vasc..

[4] Tatlidede E (2009). Resveratrol treatment protects against doxorubicin-induced cardiotoxicity by alleviating oxidative damage. Free Radic. Res..

[5] Wonders KY, Hydock DS, Schneider CM, Hayward R (2008). Acute exercise protects against doxorubicin cardiotoxicity. Integrat. Cancer Ther..

[6] Sun J (2013). Isorhamnetin protects against doxorubicin-induced cardiotoxicity in vivo and in vitro. PLoS ONE.

[7] Chen CT, Wang ZH, Hsu CC, Lin HH, Chen JH (2015). In vivo protective effects of diosgenin against doxorubicin-induced cardiotoxicity. Nutrients.

[8] Maltais S, Perrault LP, Ly HQ (2011). The bone marrow-cardiac axis: role of endothelial progenitor cells in heart failure. Eur. J. Cardio-thor. Surg. Off. J. Eur. Assoc. Cardio-thor. Surg..

[9] Michowitz Y (2007). Circulating endothelial progenitor cells and clinical outcome in patients with congestive heart failure. Heart.

[10] Valgimigli M (2004). CD34+ and endothelial progenitor cells in patients with various degrees of congestive heart failure. Circulation.

[11] Losordo DW (2007). Intramyocardial transplantation of autologous CD34+ stem cells for intractable angina: a phase I/IIa double-blind, randomized controlled trial. Circulation.

[12] Losordo DW (2011). Intramyocardial, autologous CD34+ cell therapy for refractory angina. Circ. Res..

[13] Povsic TJ (2013). A phase 3, randomized, double-blinded, active-controlled, unblinded standard of care study assessing the efficacy and safety of intramyocardial autologous CD34+ cell administration in patients with refractory angina: design of the RENEW study. Am. Heart J..

[14] Monsalve FA, Pyarasani RD, Delgado-Lopez F, Moore-Carrasco R (2013). Peroxisome proliferator-activated receptor targets for the treatment of metabolic diseases. Mediators Inflamm..

[15] Oka S (2015). Peroxisome proliferator activated receptor-alpha association with silent information regulator 1 suppresses cardiac fatty acid metabolism in the failing heart. Circ. Heart Fail..

[16] Balakumar P, Rohilla A, Mahadevan N (2011). Pleiotropic actions of fenofibrate on the heart. Pharmacol. Res..

[17] Cheng H, Xi Y, Chi X, Wu Y, Liu G (2016). Fenofibrate treatment of rats with experimental autoimmune myocarditis by alleviating Treg/Th17 disorder. Cent. Eur. J. Immunol..

[18] Walker AE (2012). Fenofibrate improves vascular endothelial function by reducing oxidative stress while increasing endothelial nitric oxide synthase in healthy normolipidemic older adults. Hypertension.

[19] Yin WH, Chen JW, Chen YH, Lin SJ (2013). Fenofibrate modulates HO-1 and ameliorates endothelial expression of cell adhesion molecules in systolic heart failure. Acta Cardiol. Sin..

[20] Jen HL (2016). Peroxisome proliferator-activated receptor alpha reduces endothelin-1-caused cardiomyocyte hypertrophy by inhibiting nuclear factor-kappaB and adiponectin. Mediat. Inflamm..

[21] Deng Y (2017). PPARalpha agonist stimulated angiogenesis by improving endothelial precursor cell function via a NLRP3 inflammasome pathway. Cell. Physiol. Biochem. Int. J. Exp. Cell. Physiol. Biochem. Pharmacol..

[22] Wang Z (2014). PPARalpha regulates mobilization and homing of endothelial progenitor cells through the HIF-1alpha/SDF-1 pathway. Invest. Ophthalmol. Vis. Sci..

[23] Swain SM (1999). Doxorubicin-induced cardiomyopathy. N. Engl. J. Med..

[24] Chen Y, Jungsuwadee P, Vore M, Butterfield DA, St Clair DK (2007). Collateral damage in cancer chemotherapy: oxidative stress in nontargeted tissues. Mol. Intervent..

[25] Kluza J (2004). Mitochondrial proliferation during apoptosis induced by anticancer agents: effects of doxorubicin and mitoxantrone on cancer and cardiac cells. Oncogene.

[26] Yao CX (2011). Effects of doxorubicin and fenofibrate on the activities of NADH oxidase and citrate synthase in mice. Basic Clin. Pharmacol. Toxicol..

[27] Jen HL, Yin WH, Chen JW, Lin SJ (2017). Endothelin-1-induced cell hypertrophy in cardiomyocytes is improved by fenofibrate: possible roles of adiponectin. J. Atherosc. Thromb..

[28] Uygur R (2014). Cardioprotective effects of fish omega-3 fatty acids on doxorubicin-induced cardiotoxicity in rats. Hum. Exp. Toxicol..

[29] Brem H, Tomic-Canic M (2007). Cellular and molecular basis of wound healing in diabetes. J. Clin. Investig..

[30] Huang PH (2012). Far infra-red therapy promotes ischemia-induced angiogenesis in diabetic mice and restores high glucose-suppressed endothelial progenitor cell functions. Cardiovasc. Diabetol..

[31] Chen JS (2011). Nrf-2 mediated heme oxygenase-1 expression, an antioxidant-independent mechanism, contributes to anti-atherogenesis and vascular protective effects of Ginkgo biloba extract. Atherosclerosis.

[32] Zhang W (2015). Loss of multidrug resistance-associated protein 1 potentiates chronic doxorubicin-induced cardiac dysfunction in mice. J. Pharmacol. Exp. Therap..

[33] Wang L (2016). Protection against doxorubicin-induced myocardial dysfunction in mice by cardiac-specific expression of carboxyl terminus of hsp70-interacting protein. Sci. Rep..

[34] McGowan JV (2017). Anthracycline chemotherapy and cardiotoxicity. Cardiovasc. Drugs Ther..

[35] Hamed S (2006). Erythropoietin improves myocardial performance in doxorubicin-induced cardiomyopathy. Eur. Heart J..

[36] Zhao Z, Luo J, Ma L, Luo X, Huang L (2015). Effect of granulocyte colony stimulating EPC on cardiac function and myocardial energy expenditure in patients with heart failure after myocardial infarction. Int. J. Clin. Exp. Med..

[37] Premer C (2015). Allogeneic mesenchymal stem cells restore endothelial function in heart failure by stimulating endothelial progenitor cells. EBioMedicine.

[38] Gutierrez FR (2008). Increased activities of cardiac matrix metalloproteinases matrix metalloproteinase (MMP)-2 and MMP-9 are associated with mortality during the acute phase of experimental Trypanosoma cruzi infection. J. Infect. Dis..

[39] Okamoto H (2005). Inhibition of NF-kappaB signaling by fenofibrate, a peroxisome proliferator-activated receptor-alpha ligand, presents a therapeutic strategy for rheumatoid arthritis. Clin. Exp. Rheumatol..

[40] Lin R (2006). Fenofibrate inhibits tumor necrosis factor-alpha-induced expression of CD40 and matrix metalloproteinase in human vascular endothelial cells. Nan fang yi ke da xue xue bao J. South. Med. Univ..

[41] Maruyama S (2011). Adiponectin ameliorates doxorubicin-induced cardiotoxicity through Akt protein-dependent mechanism. J. Biol. Chem..

[42] Nakamura T (2006). Association of hyperadiponectinemia with severity of ventricular dysfunction in congestive heart failure. Circ. J. Off. J. Jpn. Circ. Soc..

[43] Kistorp C (2005). Plasma adiponectin, body mass index, and mortality in patients with chronic heart failure. Circulation.

[44] Silva FB (2015). Hormone therapy with tamoxifen reduces plasma levels of NT-B-type natriuretic peptide but does not change ventricular ejection fraction after chemotherapy in women with breast cancer. Braz. J. Med. Biol. Res. Revista brasileira de pesquisas medicas e biologicas.

[45] Tokarska-Schlattner M, Wallimann T, Schlattner U (2006). Alterations in myocardial energy metabolism induced by the anti-cancer drug doxorubicin. C.R. Biol..

[46] Wei W (2014). Statins and fibrates do not affect development of spontaneous cartilage damage in STR/Ort mice. Osteoarthr. Cartil..

[47] Haybar H (2019). Effect of gemfibrozil on cardiotoxicity induced by doxorubicin in male experimental rats. Biomed. Pharmacother. Biomedecine pharmacotherapie.

[48] Murakami H (2006). Fenofibrate activates AMPK and increases eNOS phosphorylation in HUVEC. Biochem. Biophys. Res. Commun..

[49] Li P (2010). Fenofibrate promotes ischemia-induced revascularization through the adiponectin-dependent pathway. Am. J. Physiol. Endocrinol. Metab..

