# Supplementary material for: Fenofibrate attenuates doxorubicin-induced cardiac dysfunction in mice via activating the eNOS/EPC pathway
Source: Sci Rep. 2021 Jan 13;11:1159. doi: 10.1038/s41598-021-80984-4 (PMC7806979; doi:10.1038/s41598-021-80984-4)

**Fenofibrate attenuates doxorubicin-induced cardiac dysfunction in mice via activating the eNOS/EPC pathway**

Wen-Pin Huang, Wei-Hsian Yin, Jia-Shiong Chen, Po-Hsun Huang, Jaw-Wen Chen, Shing-Jong Lin

**Supplemental data**

**Animal experiments flow chart**

Experimental flow chart contains first part: mainly to analyze the direct effect of fenofibrate on the amount of EPC in peripheral blood, 4 mice for saline, 5 mice for fenofibrate 20 mgkg-1d-1, 5 mice for fenofibrate 100 mgkg-1d-1. Fenofibrate has no significant effect on body weight and toxicity under the feeding condition of high concentration 100mgkg-1d-1 for 4 weeks (S-fig1). The second part: mainly analyze the effect of fenofibrate on the number of peripheral blood EPC in the progression of heart failure induced by DOX, 6 mice for saline, 13 mice for saline + DOX, 13 mice for DOX + fenofibrate 100, 7 mice for DOX + fenofibrate 100 + L-NAME. The once-a-week tail vein administration of DOX (accumulatively 20 mg/kg) did not observe dramatic weight loss and a rapid recovery after drug withdrawal. When fenofibrate was administered in combination, the body weight decreased significantly with the administration time. Although no mice died, the individuals were weak, especially in the DOX + fenofibrate 100 + L-NAME group. At the expiration of the medication period, blood sampling through a face clip and fractional shortening ratios assay did not cause death.


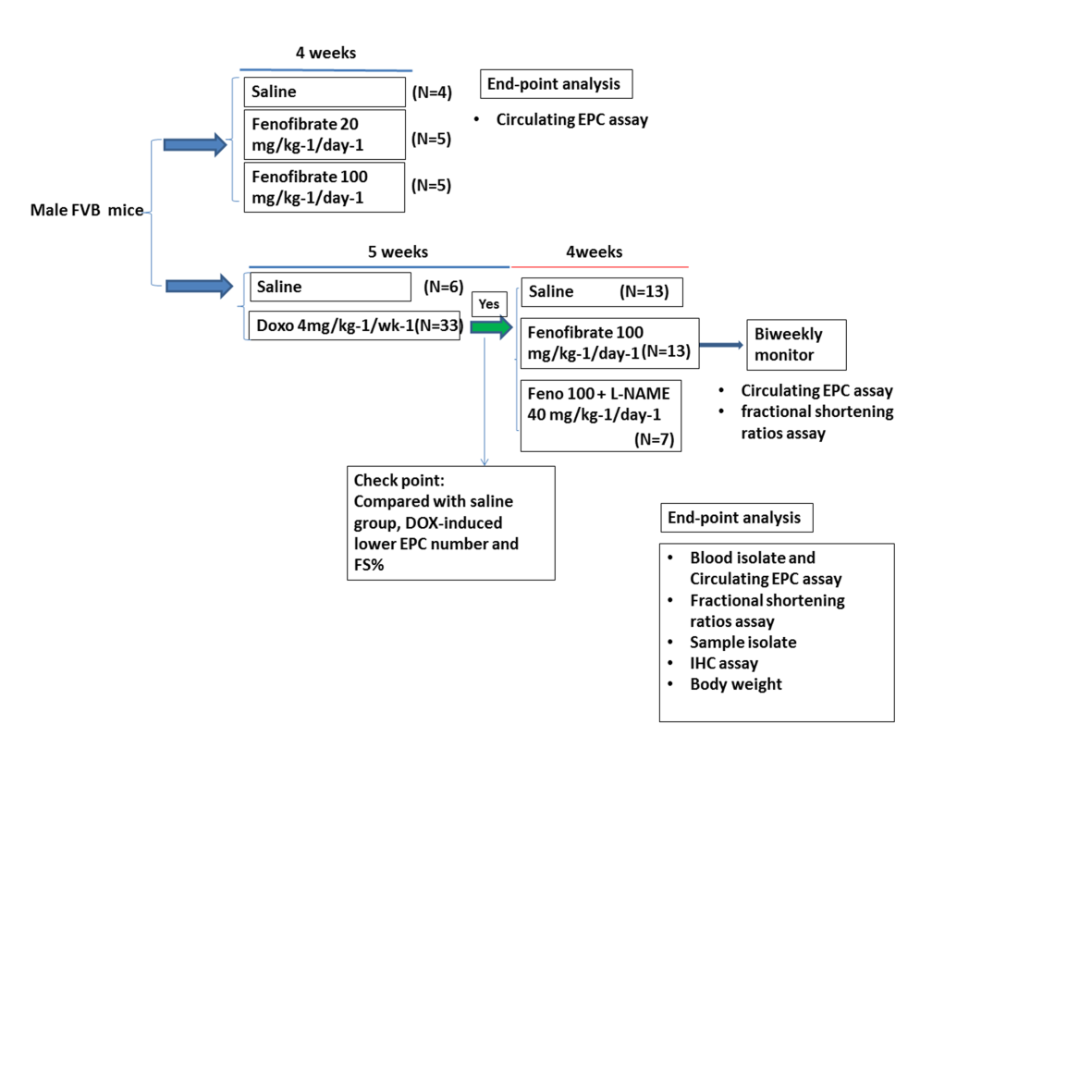


**EPC identify**

We analyzed the CD117 and CD135 by flow cytometry analysis of peripheral blood samples from wild-type mice, showing that the sca1+/flk1+ cells are only 2.4% positive for CD117 (c-kit) and approximately 1.5% positive for CD135 (Flt3) (S-fig 2). We also analyzed the T cell population cell markers. Sca1+/flk1+ cells are also a middle level about 35.3% of the T-cell co-receptor (CD3) expressed by the cells, approximately 15% express the Cytotoxic (CD8) T cells, 1.5% of T-helper (CD4), and 5% of CD16/32 (CD16/CD32 are expressed on B cells, monocytes/macrophages, NK cells, granulocytes, mast cells, and dendritic cells). Finally, we assessed the myeloid-derived cell markers Ly6C and CD11b. Sca1+/flk1+ cells appeared were 2.1% positive of Ly6C and 4.8% positive of CD11b (S-fig 3).


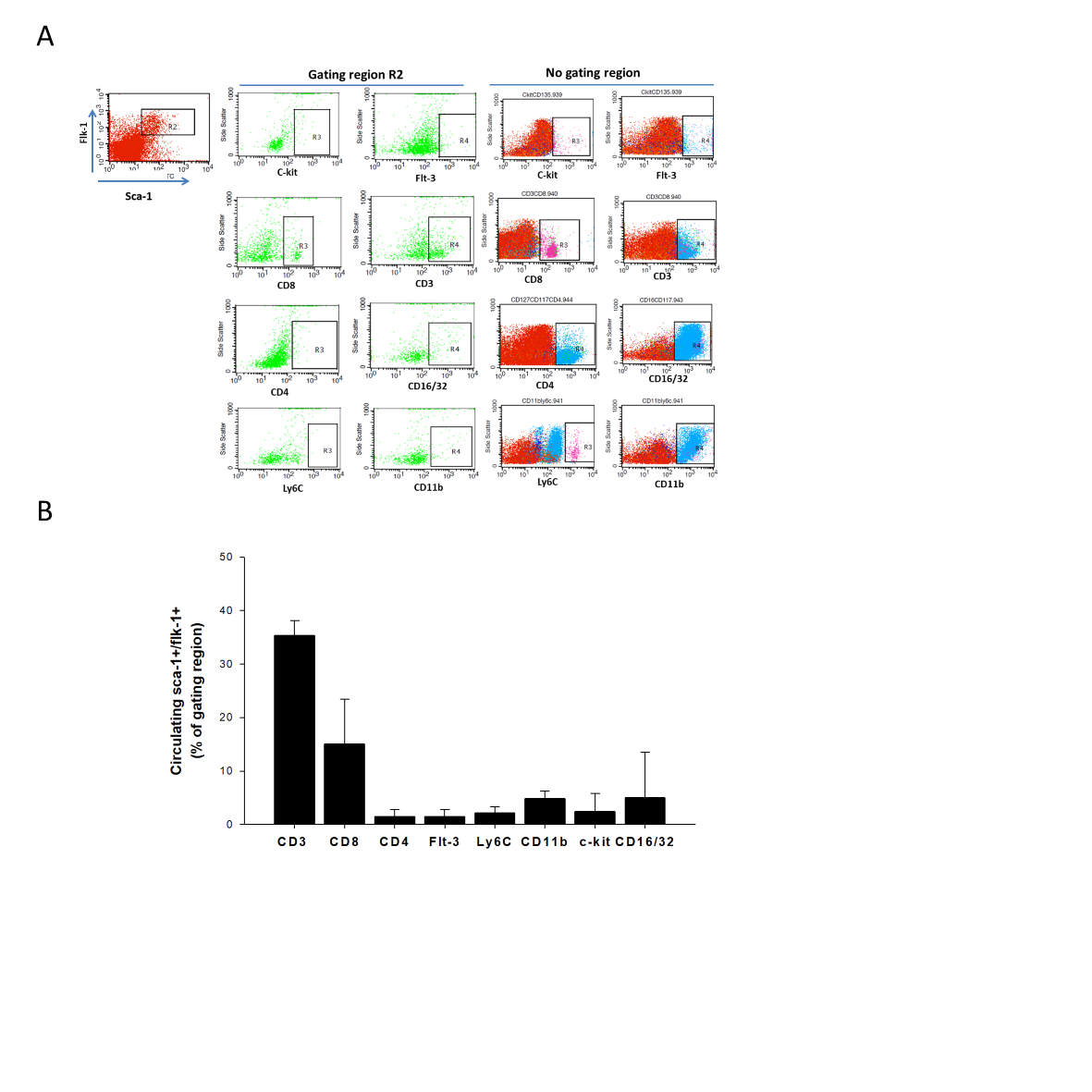

Supplement: Supplementary file 1 — Supplementary Informatipon 1. [file 41598_2021_80984_MOESM1_ESM.docx]
