# Supplementary material for: Fenofibrate attenuates doxorubicin-induced cardiac dysfunction in mice via activating the eNOS/EPC pathway
Source: Sci Rep. 2021 Jan 13;11:1159. doi: 10.1038/s41598-021-80984-4 (PMC7806979; doi:10.1038/s41598-021-80984-4)
Supplement: Supplementary file 2 — Supplementary Informatipon 2. [file 41598_2021_80984_MOESM2_ESM.pptx]

## Slide 1
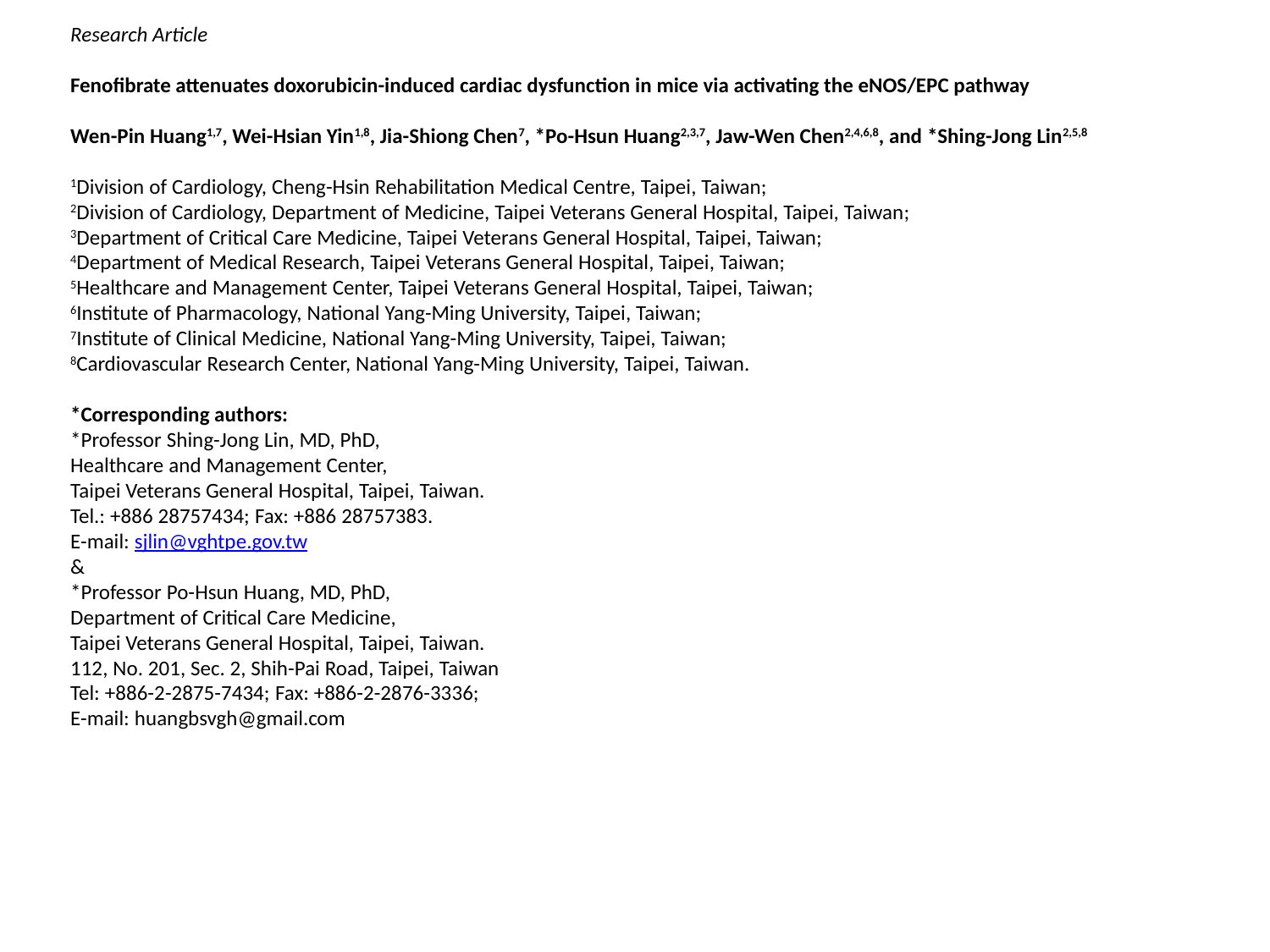

Research Article
Fenofibrate attenuates doxorubicin-induced cardiac dysfunction in mice via activating the eNOS/EPC pathway
Wen-Pin Huang1,7, Wei-Hsian Yin1,8, Jia-Shiong Chen7, *Po-Hsun Huang2,3,7, Jaw-Wen Chen2,4,6,8, and *Shing-Jong Lin2,5,8
1Division of Cardiology, Cheng-Hsin Rehabilitation Medical Centre, Taipei, Taiwan;
2Division of Cardiology, Department of Medicine, Taipei Veterans General Hospital, Taipei, Taiwan;
3Department of Critical Care Medicine, Taipei Veterans General Hospital, Taipei, Taiwan;
4Department of Medical Research, Taipei Veterans General Hospital, Taipei, Taiwan;
5Healthcare and Management Center, Taipei Veterans General Hospital, Taipei, Taiwan;
6Institute of Pharmacology, National Yang-Ming University, Taipei, Taiwan;
7Institute of Clinical Medicine, National Yang-Ming University, Taipei, Taiwan;
8Cardiovascular Research Center, National Yang-Ming University, Taipei, Taiwan.
*Corresponding authors:
*Professor Shing-Jong Lin, MD, PhD,
Healthcare and Management Center,
Taipei Veterans General Hospital, Taipei, Taiwan.
Tel.: +886 28757434; Fax: +886 28757383.
E-mail: sjlin@vghtpe.gov.tw
&
*Professor Po-Hsun Huang, MD, PhD,
Department of Critical Care Medicine,
Taipei Veterans General Hospital, Taipei, Taiwan.
112, No. 201, Sec. 2, Shih-Pai Road, Taipei, Taiwan
Tel: +886-2-2875-7434; Fax: +886-2-2876-3336;
E-mail: huangbsvgh@gmail.com

## Slide 2
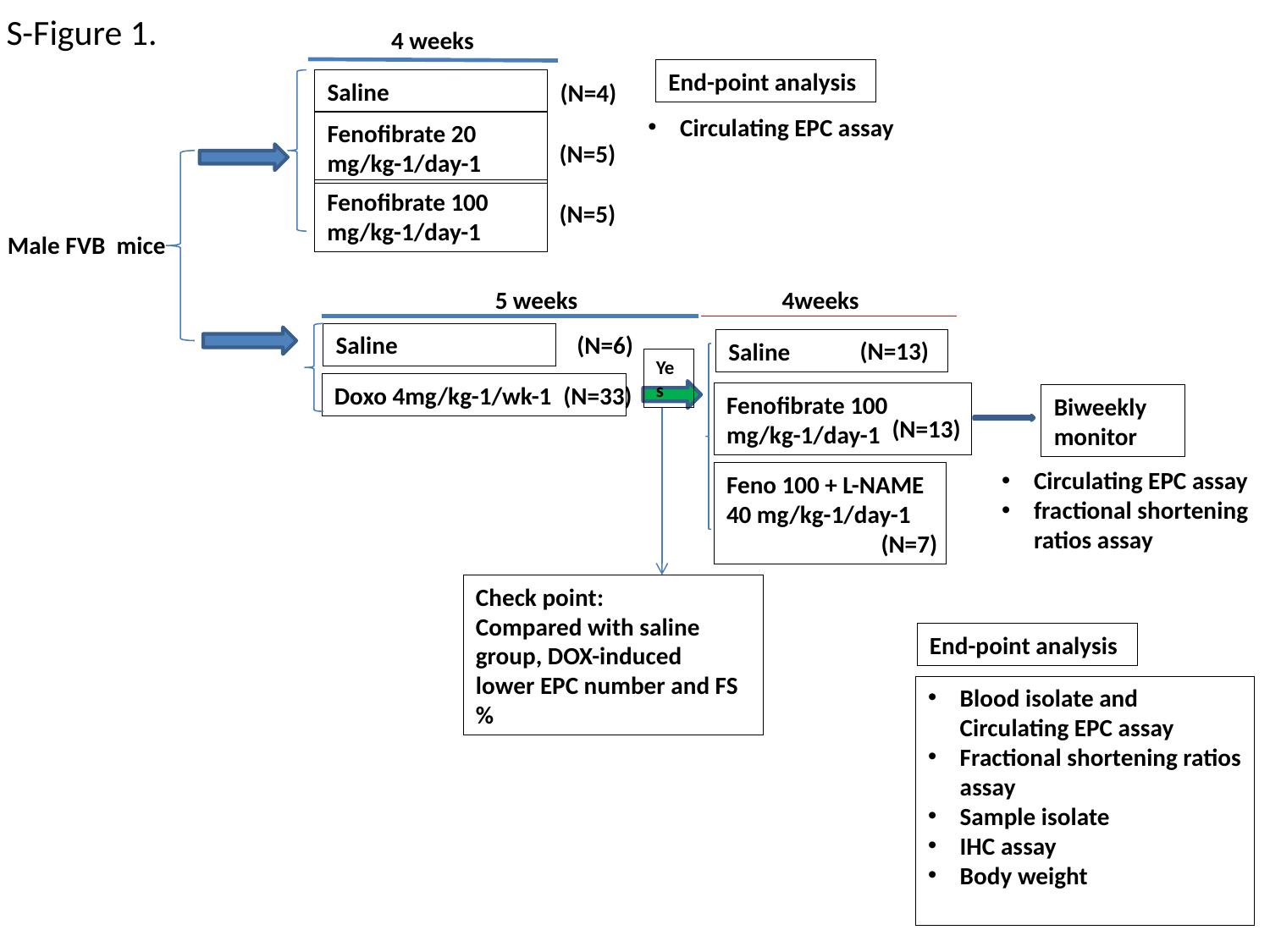

S-Figure 1.
4 weeks
End-point analysis
Saline
(N=4)
Circulating EPC assay
Fenofibrate 20 mg/kg-1/day-1
(N=5)
Fenofibrate 100 mg/kg-1/day-1
(N=5)
Male FVB mice
5 weeks
4weeks
(N=6)
Saline
(N=13)
Saline
Yes
Doxo 4mg/kg-1/wk-1
(N=33)
Fenofibrate 100 mg/kg-1/day-1
Biweekly monitor
(N=13)
Circulating EPC assay
fractional shortening ratios assay
Feno 100 + L-NAME 40 mg/kg-1/day-1
(N=7)
Check point:
Compared with saline group, DOX-induced lower EPC number and FS%
End-point analysis
Blood isolate and Circulating EPC assay
Fractional shortening ratios assay
Sample isolate
IHC assay
Body weight

## Slide 3
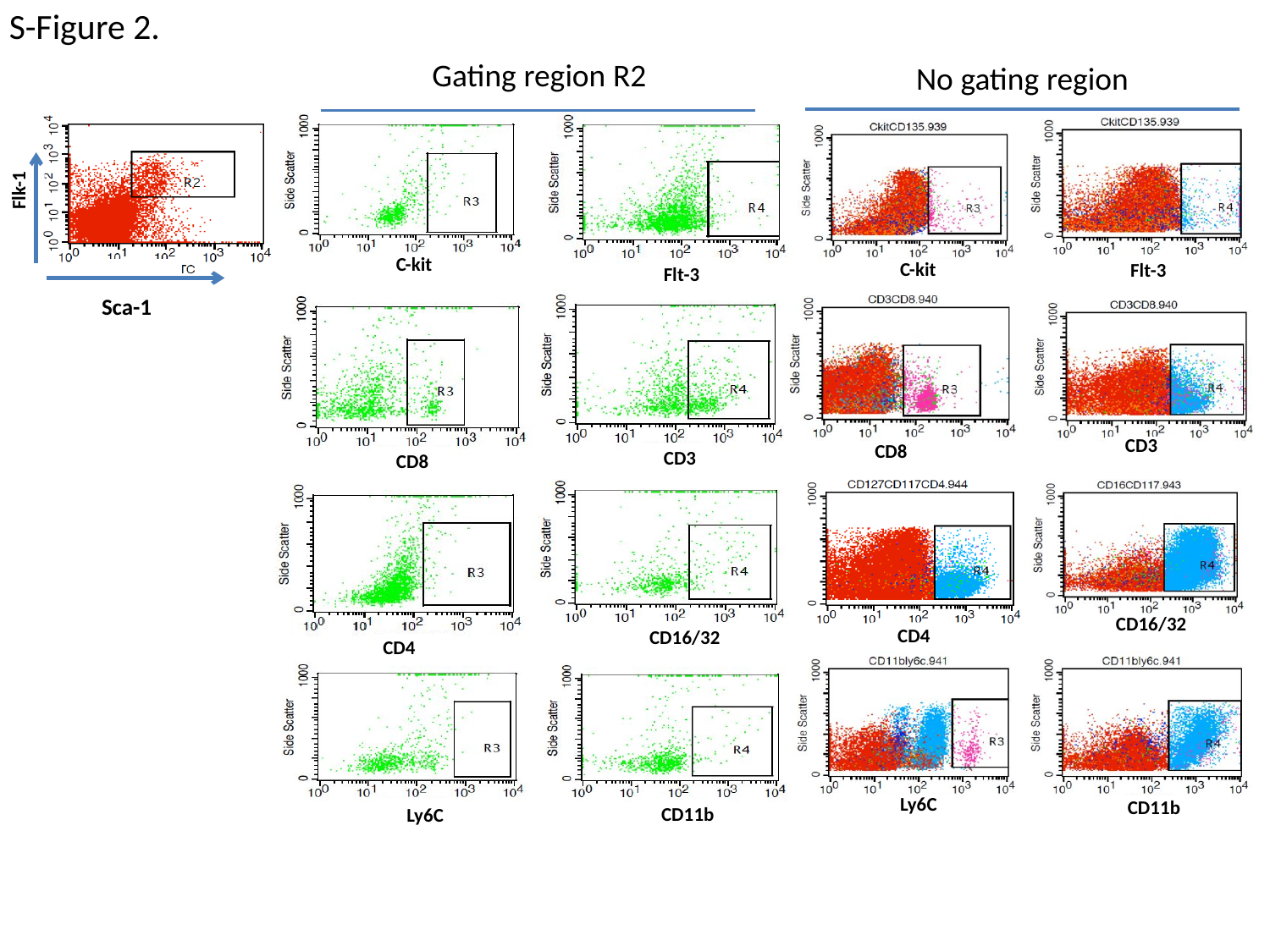

S-Figure 2.
Gating region R2
No gating region
C-kit
Flt-3
Flt-3
C-kit
Flk-1
CD3
CD8
Sca-1
CD8
CD3
CD16/32
CD4
CD4
CD16/32
C-kit
Ly6C
CD11b
CD11b
Ly6C

## Slide 4
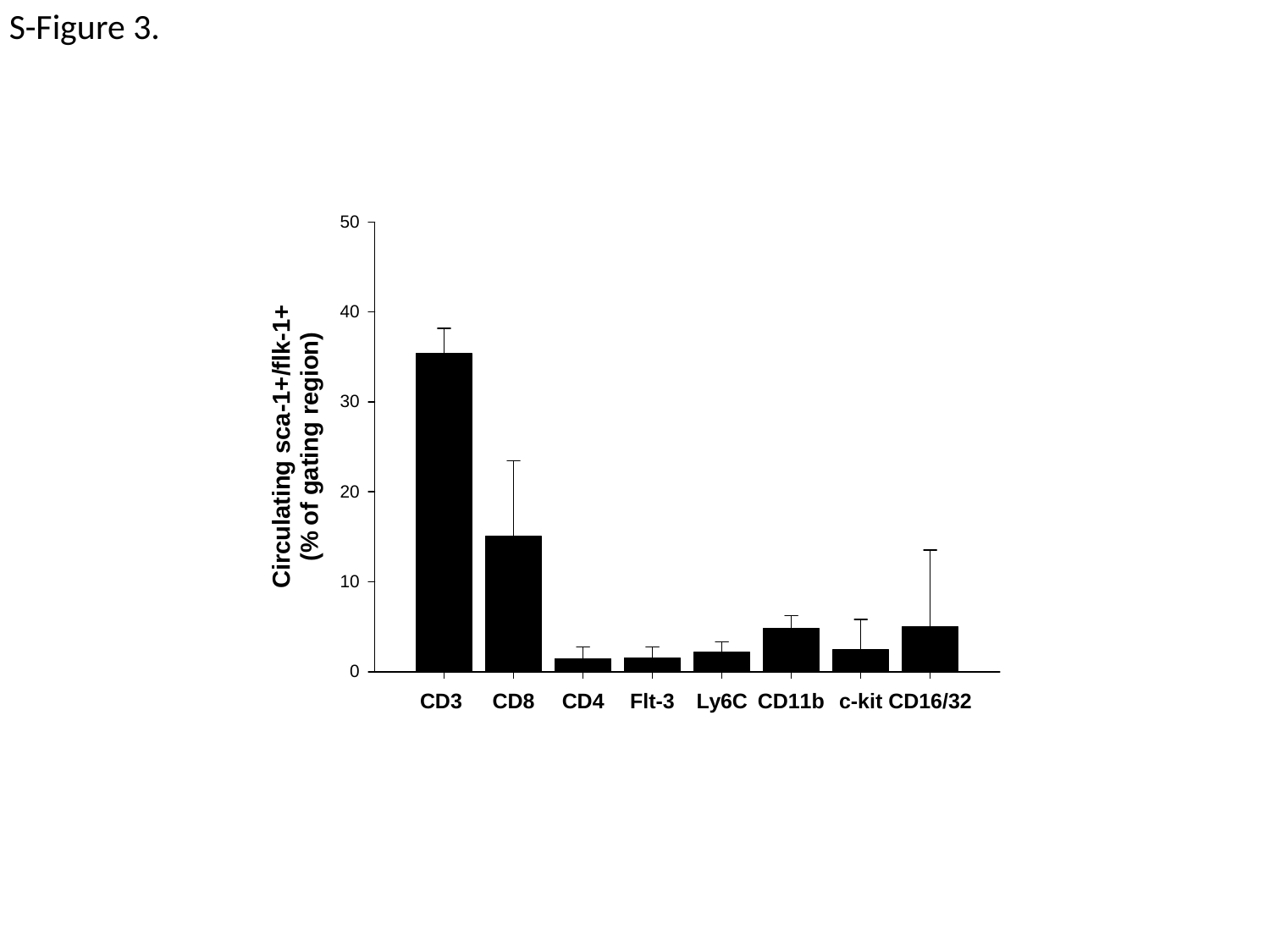

S-Figure 3.

## Slide 5
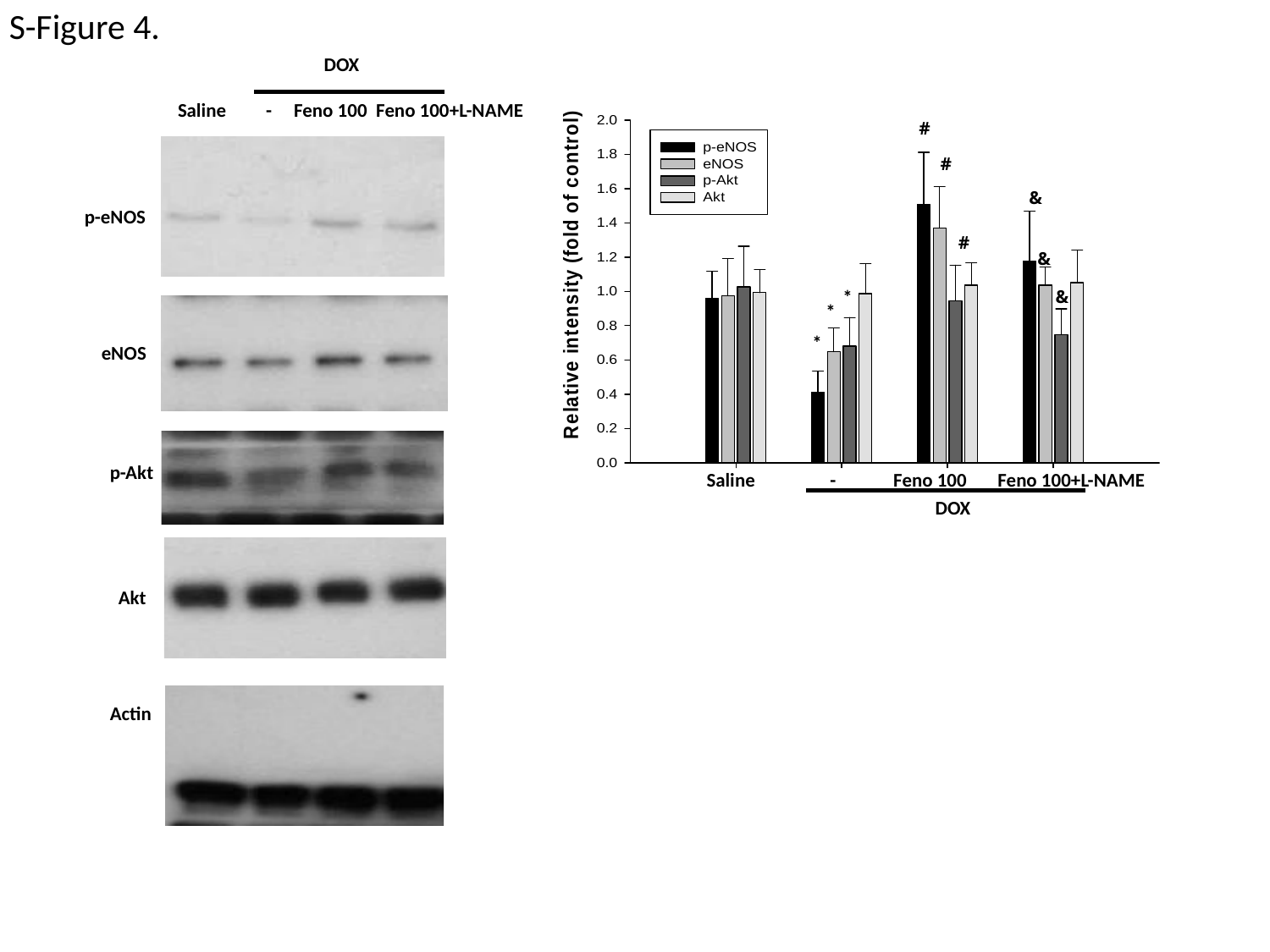

S-Figure 4.
DOX
#
#
&
#
&
*
&
*
*
 Saline - Feno 100 Feno 100+L-NAME
DOX
 Saline - Feno 100 Feno 100+L-NAME
p-eNOS
eNOS
p-Akt
Akt
Actin

## Slide 6
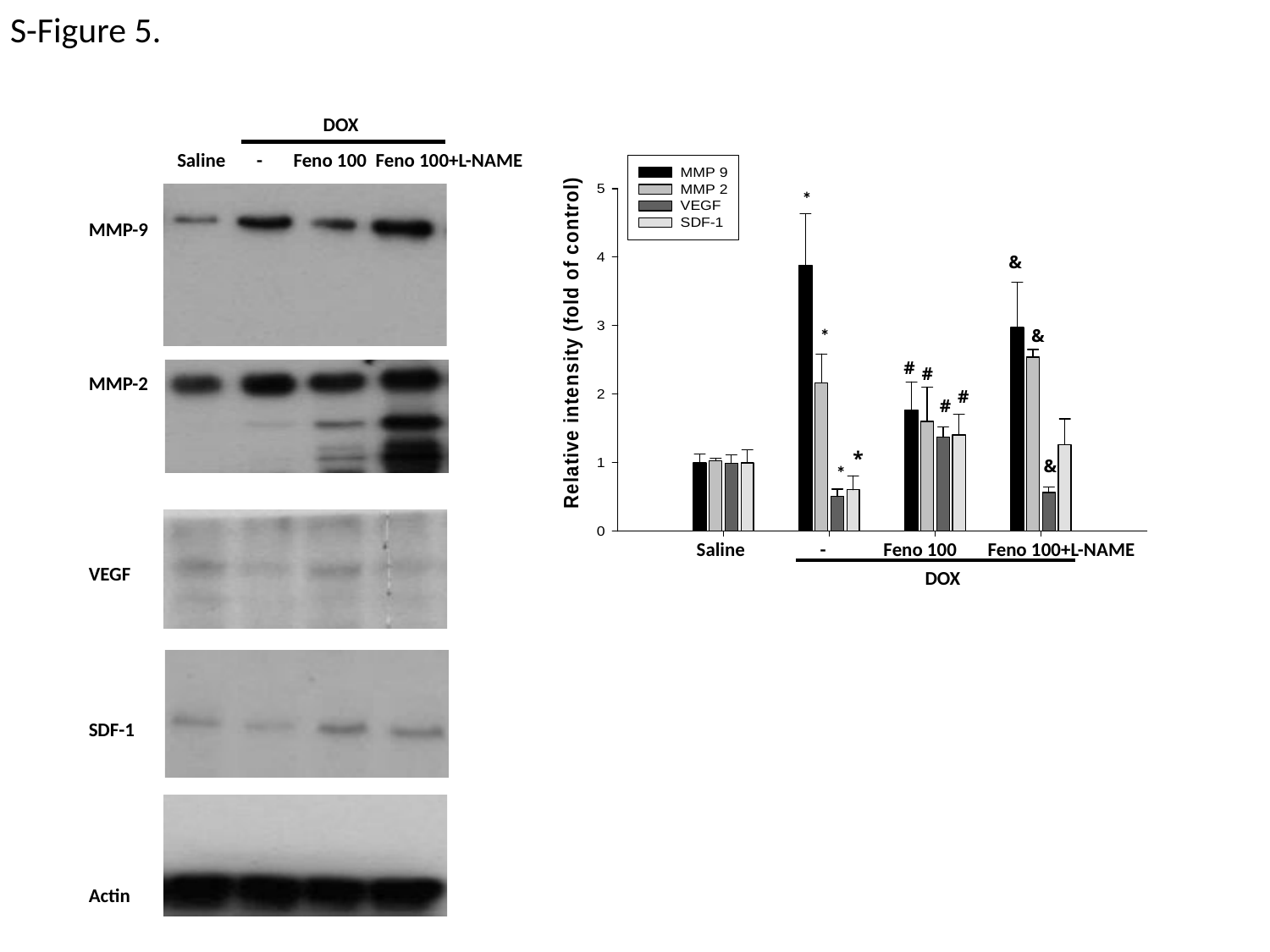

S-Figure 5.
DOX
*
&
*
&
#
#
#
#
*
&
*
 Saline - Feno 100 Feno 100+L-NAME
DOX
 Saline - Feno 100 Feno 100+L-NAME
MMP-9
MMP-2
VEGF
SDF-1
Actin
